# The impact of habitat quality inside protected areas on distribution of the Dominican Republic’s last endemic non-volant land mammals

**DOI:** 10.1093/jmammal/gyz007

**Published:** 2019-01-30

**Authors:** Rosalind J Kennerley, Malcolm A C Nicoll, Richard P Young, Samuel T Turvey, Jose M Nuñez-Miño, Jorge L Brocca, Simon J Butler

**Affiliations:** 1 Durrell Wildlife Conservation Trust, Les Augrès Manor, Trinity, Jersey, British Channel Islands; 2 Centre for Agri-Environmental Research, University of Reading, Earley Gate, Reading, United Kingdom; 3 Institute of Zoology, Zoological Society of London, Regent’s Park, London, United Kingdom; 4 Sociedad Ornithológica de la Hispaniola, Apto. 401 Residencial Las Galerías, Calle Gustavo Mejia Ricart No. 119 B, Santo Domingo, Dominican Republic; 5 University of East Anglia, Norwich Research Park, Norwich, United Kingdom

**Keywords:** Caribbean mammals, Hispaniola, hutia, indirect field signs, solenodon, systematic surveys

## Abstract

The Hispaniolan solenodon, *Solenodon paradoxus*, and Hispaniolan hutia, *Plagiodontia aedium*, are the Dominican Republic’s only surviving endemic non-volant land mammals, and are high priorities for conservation. The country has an extensive protected area (PA) network designed to maintain habitats and benefit biodiversity, but which faces significant anthropogenic threats likely to detrimentally impact both species. We examined how differences in habitats, forest structure, topography, and human activity influence presence of solenodons and hutias across the Dominican Republic. Systematic surveys of seven PAs were undertaken to record indirect signs, with presence-absence data analyzed using a multi-model inference approach incorporating ecological variables from both field and GIS data. Solenodons were detected relatively frequently, whereas detections of hutias were uncommon. Lower elevations, increased surrounding tree cover, canopy closure, and reduced levels of low vegetation are all associated with increased probability of detecting solenodons, whereas agriculture and mangrove represent poor-quality habitat. Increased canopy closure, tree basal area (indicating older-growth forest), and increased rock substrate (providing more den sites) are associated with increased probability of detecting hutias. Our findings indicated that human activities within PAs are likely to negatively affect both species, and conservation activities should focus on preventing encroachment and conversion of forest to agriculture to maintain high-quality forest habitats.

El solenodonte de la Hispaniola, *Solenodon paradoxus*, y la hutia de la Hispaniola, *Plagiodontia aedium*, son los únicos mamíferos endémicos terrestres no voladores que sobreviven en la República Dominicana, su conservación es de alta prioridad. El país tiene una extensa red de áreas protegidas (AP) diseñada para mantener hábitats y beneficiar la biodiversidad, pero se enfrenta a amenazas antropogénicas. Sin embargo, no existen datos cuantitativos para evaluar las presiones antropogénicas que amenazan a los solenodontes y las hutias. Examinamos cómo las diferencias en los hábitats, la estructura del bosque, la topografía y la actividad humana influyen la presencia de solenodontes y hutias en toda la República Dominicana. Se realizaron encuestas sistemáticas de siete AP para registrar los signos indirectos de ambas especies, los datos de presencia/ausencia fueron analizados mediante inferencia multimodelo que incorpora variables ecológicas de los datos de campo y Sistema de Información Geográfica. Los Solenodontes se detectaron relativamente frecuentemente, mientras que las detecciones de hutias fueron menos comunes. Las elevaciones más bajas, el aumento de la cubierta arbórea circundante, el cierre del dosel y los niveles reducidos de vegetación baja se asocian con una mayor probabilidad de detectar solenodones. Mientras que la agricultura y los manglares representan un hábitat de mala calidad para el solenodonte. Aumento del cierre del dosel, área basal del árbol (que indica un bosque más antiguo) y un sustrato con mayor proporcion de roca (que proporciona más sitios para madrigueras) se asocian con una mayor probabilidad de detectar hutias. Nuestros hallazgos indican que las actividades humanas dentro de las AP pueden afectar negativamente a ambas especies. Las actividades de conservación deberían enfocarse en mantener hábitats forestales de alta calidad por medio de prevenir la invasión y la conversión de los bosques a agricultura.

Establishment and maintenance of protected areas (PAs) is a common approach for preserving important regions of endemism and biodiversity. The role and benefits of PAs to conservation, when properly enforced, are well documented (Bruner et al. 2001; Rodrigues et al. 2004; Cantú-Salazar and Gaston 2010; Coetzee et al. 2014). The Caribbean is a globally important insular biodiversity hotspot, with 74% of 69 mammal species endemic to the region (Mittermeier et al. 2004; Anadón-Irizarry et al. 2012). Key Biodiversity Areas (KBAs) contain over one-half of all threatened species in the Caribbean, and 51% overlap partially or completely with PAs (Anadón-Irizarry et al. 2012).

Hispaniola, divided politically into Haiti and the Dominican Republic, is the second largest Caribbean island. The importance of the PA network in the Dominican Republic is highlighted by the fact that 18% (868,314 ha) of the country’s land area is covered by KBAs, of which 88% is either completely or partially protected; in comparison, 13% (360,314 ha) of Haiti is covered by KBAs, but only 18% is protected (Anadón-Irizarry et al. 2012). With 22% of land under strict protection (IUCN and UNEP-WCMC 2014), the Dominican Republic has among the highest percentage of PAs of any country, despite being relatively poor and densely populated (Holmes 2010). Like many tropical regions, continuing human population growth places increasing pressure on natural ecosystems, often leading to unsustainable land-use practices and damage or loss of forest habitats (Foley et al. 2005; DeFries et al. 2007). Although intensive exploitation and settlements that alter the ecosystem of PAs in the Dominican Republic are not permitted, their boundaries are permeable to encroachment and settlement (Perdomo and Arias 2008). For example, creation of infrastructure for development of scientific, recreational, and tourist activities within PAs is permissible (Ministerio de Medio Ambiente y Recursos Naturales 2004). However, a lack of knowledge of regulations and permitted uses of PAs in local communities, alongside limited enforcement, facilitates ongoing environmental degradation through resource extraction inside their boundaries, which can drive declines or extinctions of species that PAs were designated to protect (Baillie et al. 2004; Caro and Scholte 2007).

The Dominican Republic contains two surviving native non-volant land mammals, the Hispaniolan hutia (Capromyidae: *Plagiodontia aedium*), a large caviomorph rodent, and the Hispaniolan solenodon (Solenodontidae: *Solenodon paradoxus*), a eulipotyphlan insectivore. Both species always have been considered rare and threatened (Verrill 1907; Allen 1942; Fisher and Blomberg 2011) and are currently listed as Endangered by IUCN (2018). They are both also global conservation priorities based on evolutionary distinctiveness (Collen et al. 2011). Habitat loss, invasive species, persecution, and hunting all are considered potential threats (IUCN 2018), but the ecology of both species is poorly understood and available data about status, distribution, and threats are limited and contradictory. Both species are considered dependent on native forest and predominantly use limestone caves as denning sites, although hutias also reportedly use tree cavities and solenodons reportedly use fallen logs (Woods 1981; Ottenwalder 1985). Hutias are primarily arboreal and herbivorous, feeding on leaves, fruit, and bark, whereas solenodons are terrestrial, foraging in soil and leaf litter for invertebrates (Woods 1981; Sullivan 1983). Woods (1981) reported that hutias were habitat generalists, potentially making them more resilient to human pressures, whereas solenodons were more vulnerable to habitat change. Ottenwalder (1985) described both species as widely distributed, but reported that solenodon populations were highly fragmented and declining in number. Conversely, Sullivan (1983) reported drastic reductions in hutia populations and distribution associated with development and deforestation, and although the species persisted in patches of appropriate habitat, it was extremely rare.

We conducted a large-scale field survey to investigate the ecology and distribution of Hispaniola’s native land mammals to increase the conservation evidence base for both species. Herein, we use the extensive data set generated by this survey on occurrence of solenodons and hutias in seven PAs across the Dominican Republic to examine how differences in habitat type, forest structure, topography, and human activity influence presence of these species. We use our findings to provide recommendations for PA management that can benefit conservation of both species.

## Materials and Methods

### 

#### Survey sites and sampling

**Fig. 1.**
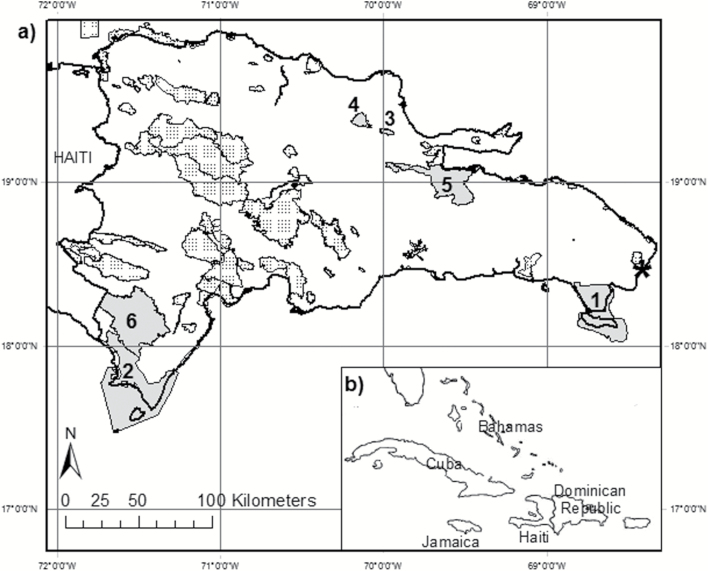
a) Map of the Dominican Republic protected area network (stippled gray), showing six surveyed National Parks (solid gray: 1, Del Este; 2, Jaragua; 3, Loma Guaconejo; 4, Loma Quita Espuela; 5, Los Haitises; 6. Sierra de Bahoruco) and one surveyed privately owned reserve (Punta Cana Ecological Reserve, asterisk). b) Location of the Dominican Republic in the western Caribbean.

Between March 2010 and June 2012, data on presence or absence of hutias and solenodons, together with several measures of fine-scale habitat structure and composition, were collected from 289 survey points across seven national parks (NPs) or privately owned PAs in the Dominican Republic, which together represent 31.2% of the area covered by the country’s PA network. Selected PAs are widely distributed, and represent a broad range of habitats, vegetation types, and topographic or climatic variables (Fig. 1). With only limited prior knowledge of the distribution of both species, we attempted to survey ≥ 15 points in each PA to capture variation in species distribution. If selected points proved unsafe to access, alternative randomly allocated points were selected.

Sierra de Bahoruco NP (1,125 km^2^, 18°10′N, 71°31′W, 300–2,367 m elevation; 168 points collected between 5 March 2010 and 20 April 2011) is a mountainous area with diverse habitats from dry broadleaf forest on lower slopes to wet broadleaf forest at higher elevations, which transitions at 1,100 m into pine forest with shallow soils. Given its high elevational and habitat variation, we invested extensive survey effort in this PA. In the absence of high-quality vegetation maps, the PA was stratified into 400-m elevational bands to ensure we surveyed all vegetation types, with ca. 20 points per stratum.

Jaragua NP (1,654 km^2^, 17°49′N, 71°32′W, 0–331 m elevation; 22 points collected between 19 July 2010 and 14 January 2011) is a lowland PA containing dry forest, mangroves, and coastal wetlands. Del Este NP (428 km^2^, 18°16′N, 68°42′W, 0–60 m elevation; 16 points collected between 6 July 2010 and 17 June 2011) is another lowland PA containing broadleaf forest, karst forest, scrub, savannah, and wetlands. In both PAs, we were able to stratify survey effort by vegetation type, and allocated points proportionally to area of each stratum and in randomly chosen locations (categories in Jaragua: low or no vegetation cover, dry scrub, dry forest, broadleaf semi-humid forest, mangrove; categories in Del Este: mangrove, semi-humid broadleaf forest, broadleaf scrub).

Los Haitises NP (634 km^2^, 19°01′N, 69°37′W, 0–287 m elevation; 40 points collected between 13 August 2011 and 23 June 2012) has irregular topography supporting tropical moist forest, karst forest, mangroves, wetlands, and coastal forest. Loma Quita Espuela Scientific Reserve (92 km^2^, 19°23′N, 70°08′W, 100–985 m elevation; 19 points collected between 11 August 2011 and 19 December 2011) contains subtropical moist forest, cloud forest, rainforest, riparian forest, and wetlands. Loma Guaconejo Scientific Reserve (23 km^2^, 19°19′N, 69°59′W, 0–606 m elevation; 19 points collected between 5 January 2012 and 11 January 2012) contains broadleaf forest, broadleaf scrub, and pasturelands. Punta Cana Ecological Reserve (11 km^2^, 18°32′N, 68°22′W, 0–15 m elevation; five points collected between 10 August 2010 and 11 August 2010) is a privately owned low-elevation PA with coastal scrub and older secondary-growth dry forest. Selection of points in these PAs was random.

#### Plot methodology

Each plot was a 20-m-radius circle (total area: 1,256 m^2^) around the survey point, within which the following variables were recorded:

#### Mammal signs

**Fig. 2.**
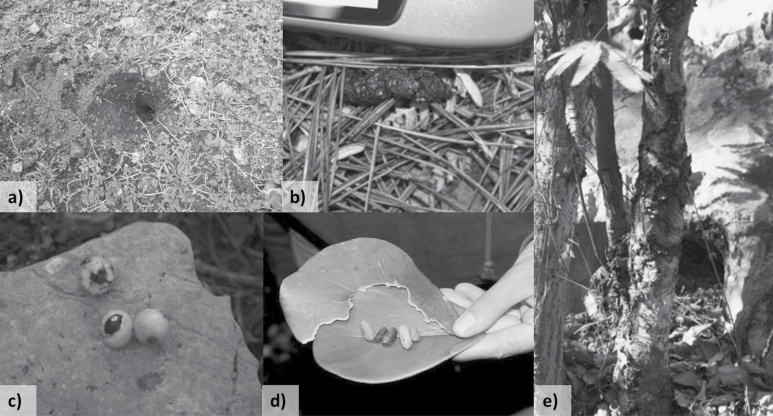
Hispaniolan solenodon (*Solenodon paradoxus*) field signs: a) conical-shaped foraging “nose-pokes”; b) feces. Hispaniolan hutia (*Plagiodontia aedium*) field signs: c) gnawed fruit; d) chewed leaf and fecal pellets (photo, Mongabay.com/Tiffany Roufs); e) gnawed bark on tree trunk.

As both target species have secretive nocturnal behaviors (Woods 1981), species presence was based solely on indirect measures, with no attempts made to survey using direct observation. All surveys were undertaken by a team of five experienced researchers. During daylight hours, two researchers spent 20 min searching for signs of each species (Fig. 2). Solenodon presence was determined by presence of distinctive conical holes (“nose-pokes”) made while foraging for invertebrates in soil or leaf litter. Hutia presence was determined by evidence of feeding or gnawing on fruit, bark, and leaves. Presence also was determined by feces, which is easily identifiable for both species (Mohr 1936–1938; Ottenwalder 1985). For other hutia species, urine marking is sometimes used to detect presence (e.g., Howe 1974); however, the Hispaniolan hutia is semiarboreal, and no urine marks were detected during surveys. Indirect signs of any age were used to confirm presence of species within survey plots, as the aim of the study was to understand presence of native mammals in different landscapes rather than fine-scale temporal habitat or resource use. Evidence of non-native mammal species was not recorded systematically.

#### Habitat measures

Dominant habitat was classified as: broadleaf forest (including dry, semi-humid, and cloud forest), pine forest, mangrove, agriculture (including plantations, pasture, cultivated areas, and areas where cultivation had ceased but signs of crop species remained present), and scrub (including open grassland not used for pasture, areas of recent disturbance with low vegetation, and dry or wet scrubland). Four 20-m transects were marked out in cardinal directions from the survey point, and at 2-m intervals along these transects we recorded whether the point fell on rock or soil, and number of vegetation touches by non-grass species in each 50-cm section of a vertical 250-cm pole; these data were used to determine percentage rockiness, and vegetation density and heterogeneity (Willson 1974). Small caves and crevices that native mammals might use for denning were not recorded as an additional parameter, because such features would be difficult to measure or assess in the survey plot. Canopy closure was measured at 10 m along each transect using a canopy-scope (a 25-dot array on a transparent screen held vertically 20 cm from observer—Brown et al. 2000), and calculated as the mean percentage of points where sky was not visible (with 100% representing complete canopy closure). Relative biomass was calculated as mean basal area of 10 trees with > 10 cm circumference at breast height closest to plot center.

#### Remotely sensed and derived data

ArcMap 10 was used (ESRI 2015). Point elevation was extracted from a 30-m resolution ASTER Global Digital Elevation Model (DEM—METI and NASA 2011). Distance to nearest sealed road was calculated from road data obtained from DIVA-GIS (Hijmans et al. 2004), accounting for topographical variation rather than Euclidean distance; detailed data on human settlements across the Dominican Republic are not available, so this was used as a proxy measure of likely human disturbance and isolation (Blake et al. 2007; Hickey 2012). A metric of surrounding forest cover was calculated based on 30-m resolution tree cover data from 2000 (Hansen et al. 2013), which quantifies canopy closure for all vegetation > 5 m in height; the percentage of cells around each point with > 75% canopy cover was calculated for a given species home range, using mean home-range estimates for each species to calculate cell search radius (hutia: 184 m [*n* = 12]; solenodon: 451 m [*n* = 16]—Kennerley 2014).

#### Data analysis

The influence of local and landscape-scale characteristics on probability of detecting signs of solenodons or hutias was explored using generalized linear mixed models (GLMMs) with a binomial error structure (presence = 1, absence = 0) and a logit-link, with “PA identity” included as a random effect, with separate analyses for each species. Points in Loma Quita Espuela and Loma Guaconejo were excluded from the hutia model because no signs of this species were found in any plots or when travelling between points in these PAs. Individual plots also were excluded from analyses if data for any explanatory variables were unavailable, resulting in 234 and 269 points for hutia and solenodon models, respectively.

For each species, a global model including all local and landscape-scale variables was fitted (Table 1), before a model set of all possible sub-models, ranked by Akaike’s Information Criterion corrected for small sample size (AIC_c_), was generated. For the hutia model, the total number of vegetation touches for the entire 250-cm pole height (“veg250”) was used to describe vegetation structure, to reflect the arboreal nature of this species; for the solenodon model, only the number of vegetation touches in the bottom section of the pole 50 cm above ground level (“veg50”) were considered important for this terrestrial species. Only main effects with no interaction terms were included, although “rockiness” also was included as a quadratic term for both species, because some rockiness might be necessary to provide denning sites, but extensive rockiness could result in low soil availability and therefore insufficient invertebrates for solenodons and fewer trees for hutias. Correlations between all pairs of potential explanatory variables were considered, with no evidence of strong collinearity (*r* < 0.34 in all cases). All input variables were scaled to a mean of zero and *SD* of 0.5 to allow direct comparison (Schielzeth 2010). Pseudo-*R*-squared values were determined for global GLMMs, where the marginal *R*^2^ represents variance explained by fixed factors (*R*^2^_GLMM (m)_) and the conditional *R*^2^ is interpreted as variance explained by both fixed and random factors (*R*^2^_GLMM (c)_—Nakagawa and Schielzeth 2013). To account for model uncertainty, coefficients were averaged across all models with ∆AIC_c_ ≤ 2, including zeroes as coefficients when variables were not included in particular models (Burnham and Anderson 2002). The relative importance of each predictor was calculated as the summed posterior Akaike weight of models containing that predictor which were included in the averaged model set (Burnham and Anderson 2002). All statistical analyses were performed in R v3.3.2 (R Development Core Team 2016), using lme4 (Bates et al. 2012) and MuMIn (Barton 2013).

**Table 1.** Descriptions of variables used in models to explain occurrence of Hispaniolan hutias (*Plagiodontia aedium*) and Hispaniolan solenodons (*Solenodon paradoxus*) across protected areas (PAs) in the Dominican Republic.

Explanatory variables	Description	Reason for inclusion
protected area	(1) Del Este; (2) Jaragua; (3) Loma Guaconejo; (4) Loma Quita Espuela; (5) Los Haitises; (6) Sierra de Bahoruco; (7) Punta Cana	Included as a random term in all models, because PA was a sampling unit with different survey stratification in different sites, and with nonindependence of locations. Hutia model excluded PAs (3) and (4)
canopy	Amount of canopy (%); 0% completely open to 100% completely closed	Solenodons and hutias associated with older undisturbed forest (Woods 1981)
rockiness	Measure of rockiness (%)	Caves in rocks provide denning sites for both solenodons (Ottenwalder 1985) and hutias (Woods 1981)
tree basal area	Mean tree basal area of the 10 closest trees to the survey point with a circumference > 10 cm and within the plot (m^2^)	Both species thought to be associated with older-growth forest, represented by larger tree basal areas (Woods 1981)
elevation	Elevation from sea level (m)	Conditions are less favorable for solenodons at higher elevations (Ottenwalder 1985); hutias can be found at most elevations (Woods 1981), but habitats at high elevations might provide poorer-quality diet (Sullivan 1983)
distance to road	Distance from the nearest significant road or track (m)	Both species are thought to be negatively affected by human presence due to persecution and increased threat from dogs and cats associated with people (Woods 1981; Sullivan 1983; Ottenwalder 1985; Turvey et al. 2014)
veg250	Total number of vegetation touches in all sections of the 250-cm pole	Increased vegetation provides more food for hutias (included in hutia model only)
veg50	Total number of vegetation touches in the first 50 cm above the ground	Vegetation could affect soil conditions and therefore the invertebrate prey available to solenodons (included in solenodon model only)
tree cover (hutia)	Based on the 2000 tree cover data, percentage of cells within a 184-m radius (mean diameter of hutia home range) with > 75% tree cover	Hutias are sensitive to disturbance and degradation or fragmentation of natural habitat (Sullivan 1983) (included in hutia model only)
tree cover (solenodon)	Based on the 2000 tree cover data, percentage of cells within a 451-m radius (mean diameter of solenodon home range) with > 75% tree cover	Solenodons are associated with older undisturbed forest (Woods 1981) (included in solenodon model only)

## Results

### 

#### Hutia

Hutia signs were recorded at only 14 points across Del Este, Jaragua, Sierra de Bahoruco, and Punta Cana. Signs were recorded from 15 to 2,019 m, but only in broadleaf and rarely pine forest (Table 2). Data from Los Haitises were included in analyses although hutia signs were not found within plots, because local hutia presence was indicated by signs observed outside plots. The global model had *R*^2^_GLMM (m)_ = 0.61 and *R*^2^_GLMM (c)_ = 0.70, indicating good fit, therefore strong likelihood of model-averaging outputs providing high explanatory power. The total model set comprised 192 models, with five models considered highly plausible (ΔAIC_c_ ≤ 2; Table 3). All models in this subset included canopy closure, rockiness, and tree basal area, with the top-ranking model including these three variables exclusively. The other four explanatory variables (rockiness^2^, veg250, elevation, distance to road) received weaker support, with each only appearing in one of the top five models. Probability of detecting hutia signs increased with increasing canopy closure, tree basal area, and amount of rockiness, but decreased with increasing vegetation density, distance from nearest road, and elevation. The rockiness^2^ term was negative, indicating that although probability of detecting hutia signs increased with rockiness, this had a lessened effect at extreme levels of rockiness.

**Table 2.** Summary of plots by habitat classification showing number and percentage of plots where Hispaniolan solenodons (*Solenodon paradoxus*) and Hispaniolan hutias (*Plagiodontia aedium*) were present. Plots in Loma Quita Espuela and Loma Guaconejo excluded for hutia (see text for details).

	Solenodon			Hutia		
Habitat type	Plots	Plots with species	%	Plots	Plots with species	%
broadleaf	122	57	46.7	98	12	12.2
pine	104	31	29.8	104	2	1.9
mangrove	10	0	0.0	10	0	0.0
agriculture	32	0	0.0	24	0	0.0
scrub	21	4	19.0	15	0	0.0
TOTAL	289	92	31.8	251	14	5.6

**Table 3.** Results of model selection and model-averaging procedures for explaining occurrence of Hispaniolan hutias (*Plagiodontia aedium*) at plots (*n* = 234) across seven protected areas in the Dominican Republic; plots with missing data for any explanatory variable excluded from analyses. Models ranked in order of increasing AIC_c_ differences (∆AIC_c_); *K* = number of parameters in each model. Model-averaged regression coefficients (β; ± 95% *CI*) are averages of β_*i*_ across all models with ∆AIC_c_ ≤ 2, weighted by each model’s Akaike weight *w*_*i*_. Calculations for β include β_*i*_ = 0 when variables not present in given model. *SE* = standard error of β. *w*_*ip*_ = relative variable importance (sum of *w*_*i*_ across all models including that variable).

	Model rank					Model average		
Variable	1	2	3	4	5	β	*SE*	*w*_*ip*_
canopy	•	•	•	•	•	2.91 (0.69, 5.13)	1.13	1.00
rockiness	•	•	•	•	•	2.88 (0.46, 5.30)	1.23	1.00
tree basal area	•	•	•	•	•	2.10 (0.58, 3.61)	0.77	1.00
(rockiness)^2^		•				−0.34 (−2.53, 1.84)	1.11	0.19
elevation			•			−0.22 (−1.72, 1.28)	0.76	0.18
veg250				•		−0.11 (−1.68, 1.45)	0.79	0.14
distance to road					•	−0.05 (−0.71, 0.61)	0.34	0.14
∆AIC_c_	0	1.25	1.40	1.90	1.92			
*K*	4	5	5	5	5			
*w*_*i*_	0.36	0.19	0.18	0.14	0.14			

#### Solenodon

Solenodon signs were detected in 89 plots across all seven PAs, across a wide elevational gradient (13–2,026 m) and in broadleaf forest, pine forest, and scrub (Table 2). The global model indicated a low level of model fit to the data, with *R*^2^_GLMM (m)_ = 0.29 and *R*^2^_GLMM (c)_ = 0.61. The total model set was reduced to five models in the ∆AIC_c_ ≤ 2 subset (Table 4). Canopy closure, elevation, tree cover, and veg50 appeared in all of the top five models, with the top-ranking model containing these parameters plus distance to road and tree basal area. Probability of detection increased with greater canopy closure and higher tree cover in the wider landscape, but decreased with increasing elevation and density of low-level vegetation. Of those predictors receiving weaker support, probability of detection increased with increasing tree basal area and distance to nearest road but declined with increasing rockiness; the negative quadratic term indicates a greater rate of reduction in probability at extreme levels of rockiness.

**Table 4.** Results of model selection and model-averaging procedures for explaining occurrence of Hispaniolan solenodons (*Solenodon paradoxus*) at plots (*n* = 269) across seven protected areas in the Dominican Republic; plots with missing data for any explanatory variable excluded from analyses. Table arrangement and variables as in Table 3.

	Model rank					Model average		
Variable	1	2	3	4	5	β	*SE*	*w*_*ip*_
canopy	•	•	•	•	•	1.04 (0.30, 1.78)	0.38	1.00
elevation	•	•	•	•	•	−2.84 (−3.98, −1.70)	0.60	1.00
tree cover	•	•	•	•	•	0.79 (0.02, 1.56)	0.39	1.00
veg50	•	•	•	•	•	−1.15 (−2.18, −0.11)	0.53	1.00
distance to road	•	•	•		•	0.54 (−0.20, 1.29)	0.38	0.85
tree basal area	•		•	•	•	0.47 (−0.32, 1.26)	0.40	0.78
rockiness			•		•	−0.01 (−0.55, 0.55)	0.28	0.29
(rockiness)^2^					•	−0.14 (−1.00, 0.73)	0.44	0.13
∆AIC_c_	0	0.93	1.59	1.60	1.91			
*K*	6	5	7	5	8			
*w*_*i*_	0.34	0.22	0.16	0.15	0.13			

## Discussion

We used systematic surveys and quantitative analyses to investigate habitat associations of Hispaniola’s two surviving endemic non-volant land mammals. Both solenodons and hutias were more common at lower elevations and sites with increased canopy closure and larger trees, suggesting they require older, more pristine forest. Differences in response to landscape- or site-level features (e.g., surrounding tree cover, rockiness) are likely to reflect ecological differences between the species in diet and environmental requirements. Hutia signs were detected at far fewer sites, indicating this species might be more patchily distributed (lower area of occupancy) or have a reduced current-day geographic range (lower extent of occurrence) than solenodons. Global model fit indicated that results for hutias are robust, but results for solenodons must be interpreted with more caution.

A common problem in studies of species occurrence is the ability to interpret analyses when uncertainty exists over detection (Hirzel et al. 2006), which is often exacerbated for rare or cryptic species (Gibson et al. 2007). We are confident there was a low probability of positive detection bias for either species, because indirect signs are distinctive and could not be confused with other species (Mohr 1936–1938; Ottenwalder 1985). The potential for false negatives is more likely, and could occur for two reasons. First, animals might use plots but not leave signs, for example, if they move through sites but do not use them for feeding or denning; such false negatives might vary between species if they defecate at different rates while moving through sites. This is a particular issue with highly mobile species (Thompson 2004). However, hutias and solenodons are both thought to be central-place foragers with relatively static home ranges (Woods 1981; Sullivan 1983; Ottenwalder 1991), reducing the risk of false negatives. Second, animals could be active in plots, but either signs may not be detected, or characteristic behaviors are not consistently associated with production of signs (Gu and Swihart 2004). One method to reduce such errors is to undertake repeated measures of plots (MacKenzie and Royle 2005). Unfortunately, this was not feasible in this study because of time constraints, logistical challenges, and field conditions. However, basing our analyses on field signs rather than direct observations counters these issues to some extent. Hutias and solenodons live and move around in close family groups (Woods 1981; Sullivan 1983; Woods and Ottenwalder 1992), so signs might be expected to be relatively numerous and more detectable if they are using an area. Furthermore, signs of both species persist well under all weather conditions, therefore effectively representing a cumulative record of presence over several weeks: solenodon nose-pokes last for ca. 2 weeks; hutia and solenodon feces lasts for > 2 weeks in non-enclosed (i.e., non-cave) environments; and hutia gnawing and other feeding signs are evident over much longer periods (Hoy 2011; R. J. Kennerley, pers. obs.). To further reduce between-site variation in detectability and minimize the risk of false negatives, standardized surveys were conducted by the same team of skilled field biologists familiar with both species, with a relatively small survey area (1,256 m^2^) searched intensively for 20 min. Nonetheless, we recognize that negative effects of increasing vegetation density (veg50 for solenodon, veg250 for hutia) on probability of detecting signs of both species and the negative effect of increasing rockiness of likelihood of detecting solenodon signs could be at least partly indicative of reduced detectability of sign in these areas. Signs recorded for both species were predominantly evidence of foraging; thus, any identified habitat associations probably are more closely associated with selection of foraging habitat rather than den sites. Habitat requirements for these different activities might differ in both species, and the influence of the spatial distribution of foraging and den sites, as well as population density, on species’ detection needs further exploration.

The explanatory variables used in this study were chosen based on hypotheses derived from the limited literature available on Hispaniolan mammal ecology. Most of these variables were measurable in the field, with additional remotely sensed and derived data also used. As data were unavailable regarding human settlements across the Dominican Republic, distance to nearest significant road was used as a proxy for anthropogenic activity, as presence of a road can make nearby land easier to access and hence more likely to contain human settlements and resulting habitat modification (Beever et al. 2003; Benítez-López et al. 2010). The most recent available tree cover data for the Dominican Republic are from 2000, making it possible that patterns of landscape-level forest cover could have changed by the time fieldwork was conducted in 2010–2012. We may also not have identified all key factors affecting native species distribution to include in our analyses; for example, presence of invasive mammals such as black rats (*Rattus rattus*), mongooses (*Herpestes javanicus*), or feral cats and dogs could represent a competitive or predation threat strong enough to displace native mammals from human-modified landscapes and perhaps even areas of good habitat (Sullivan 1983; Turvey et al. 2014, 2017). However, data quantifying such threats across the Dominican Republic are currently unavailable.

### 

#### Hutia

Woods (1981) reported hutias occurred from sea level to 2,000 m; we recorded hutias across a similar elevational range. However, our models indicate that likelihood of hutia presence declines with increasing elevation. Although hutias are known to feed on a wide variety of plant species (Sullivan 1983; Woods and Ottenwalder 1992), higher elevations may contain fewer suitable food plants as vegetation changes from broadleaf to pine forest. The strong positive relationship between increasing rockiness and presence of hutias also is consistent with previous suggestions that existence of suitable cavities for den sites is the most important requirement for good-quality hutia habitat (Woods 1981; Sullivan 1983). However, our data suggested that beneficial effects may decline at extreme levels of rockiness; if rock is the dominant substrate, quantity or quality of foraging habitat may decline to a level that excludes hutias, potentially due to fewer or less palatable trees being present. The mechanism underpinning the weak, counterintuitive observation that hutia signs are more likely to be recorded closer to roads is unclear and requires further investigation.

Hutias previously were reported from numerous habitats across Hispaniola, including dry subtropical, humid broadleaf, pine, swamp, and floodplain forests (Sullivan 1983). Here, hutias only were recorded in broadleaf and pine forest, with no signs of presence in mangrove, agriculture, or scrub. Our findings show that hutias are particularly associated with old-growth forest, with increased canopy closure, and tree basal area associated with higher detection probabilities. It is unclear whether this contradiction with previously reported habitat associations represents a genuine contraction in distribution, but given that our study represents the most extensive and robust survey for hutias in Hispaniola, any absence from habitats from which they have previously been reported is cause for concern.

#### Solenodon

We detected solenodons across a wide elevational gradient (13–2,026 m), including in high-elevation pine forest, which is only present above 1,100 m in the Dominican Republic. These results are consistent with Ottenwalder (1985), who reported that solenodons occur mainly at elevations below 1,000 m but can occur up to at least 1,500 m. Higher-elevation environments containing pine forest might be less favorable for solenodons because of cooler climate, poorer soils, and moisture constraints. These characteristics are associated with lower prey availability, while requiring more energy because of cooler environments (Ottenwalder 1985).

Wet mangrove is not suitable habitat for a species that forages in soil, but low detection of solenodon signs in agriculture is more intriguing, particularly as it contradicts previous reports that farmers in the Dominican Republic regard solenodons as common (Woods 1981). As with hutias, it is not possible to confidently interpret the cause of this apparent reduction in utilized habitats identified by our survey relative to reports from the 1980s. It is possible that the range of solenodons has contracted and the species has become largely or completely restricted to forest habitats as a consequence of changes in scale and intensity of farming in the Dominican Republic (Turvey et al. 2017). The area of land under agriculture has increased, and the types of crops grown and associated management practices have changed during recent decades (Bravo-Ureta and Pinheiro 1997; Raynolds 2002; González et al. 2009). Agricultural activities are known to significantly decrease soil fertility in the Dominican Republic (Templer et al. 2005), and absence of solenodons from farmland plots could be a consequence of knock-on effects of reduced soil fertility and changing landscape and crop structure on abundance and availability of potential solenodon prey species. The positive relationship between presence of solenodons and increasing distance from roads also could reflect presence of better-quality habitat further from this index of human disturbance, or because such areas may have reduced levels of other human-associated threats such as domestic and feral dogs and cats or other potentially harmful invasive species. Previous research suggests that dogs pose a particularly significant predation threat to both hutias and solenodons, and both species are persecuted as perceived crop pests and also occasionally still hunted for food (Sullivan 1983; Ottenwalder 1991; Turvey et al. 2014).

Secondary regrowth may represent a potentially suitable habitat, as solenodons were detected reasonably regularly (19% frequency) in scrub habitat. Abandoned agricultural land in the Dominican Republic can quickly become reforested with native vegetation, and forest soil properties and processes become similar to those of undisturbed old forest sites after only a short period (Martin et al. 2004; Templer et al. 2005).

Probability of detecting solenodon signs increases with increasing tree basal area, canopy cover, and percentage tree cover in the surrounding landscape, supporting previous reports that their presence is associated with good-quality forest (Woods 1981). Solenodons den predominantly in rock clefts (Ottenwalder 1991), but our data indicated that increasing percentage of rockiness reduced the likelihood of their presence. Their main prey are invertebrates found in soil or leaf litter (Woods and Ottenwalder 1992; Ottenwalder 1999), and presence of sufficient soil for foraging is likely to be important in determining occurrence of solenodons, with increased levels of rockiness reducing prey availability and foraging opportunity. Reduced probability of recording solenodon signs in plots with denser vegetation could reflect reduced sign detectability, but also could arise because thick vegetation at ground level can reduce prey abundance and hinder access to soil invertebrates (Ottenwalder 1985).

#### Conservation implications

Ottenwalder (1985) considered that hutias were widespread in the Dominican Republic, and our survey data indicate that overall this pattern remains true, with hutias detected in several large PAs with no evidence of overall geographical contraction in extent of occurrence compared to previous distribution estimates (e.g., Sullivan 1983). However, the limited frequency of hutia detections in our study, which covered a substantial area of the Dominican Republic’s PA network, suggests that this species is rare and localized in the country. Although this apparent rarity could represent a recent decline in population size (although not in overall range extent), Sullivan (1983) also noted a lack of evidence of hutia occurrence in areas of apparently suitable and undisturbed habitat, and suggested that the species already was rare by the early 1980s. Any decline in hutia populations might therefore represent a historical rather than recent event, potentially even associated with older human-caused disappearances of other now-extinct Hispaniolan endemic mammals (Turvey 2009). Whereas hutias appear to be more numerous than solenodons in the Massif de la Hotte, Haiti (Turvey et al. 2008), recent genetic work has demonstrated that effective population sizes of hutias are much smaller in the Dominican Republic (Brace et al. 2012; Turvey et al. 2016). Two of the smaller PAs were excluded from analysis of hutias because no signs of the species were found, indicating there may be a minimum patch size requirement that we have not yet explored; thus, scale of habitat fragmentation might be an important factor in determining presence or persistence of hutias.

Solenodons previously were thought to be more threatened than hutias, with populations considered to be highly fragmented and declining in number, despite reports from farmers that the species can be locally common (Woods 1981; Ottenwalder 1985). Our results indicate that solenodons are in fact widespread and reasonably frequently detected (32% frequency) across the areas that we surveyed. This result may represent a genuine increase in distribution or abundance, or alternately that the thorough systematic methodology used in our study provided a more accurate assessment of the distribution of solenodons across the Dominican Republic than in previous studies.

The Dominican Republic has an extensive NP network (Holmes 2010), but due to poor enforcement and inadequate regulation of activities within their boundaries, these PAs are experiencing anthropogenically driven degradation of biodiversity, notably due to deforestation and increasing human settlement (Perdomo and Arias 2008; Pasachnik et al. 2016). Monitoring whether PAs continue to provide the necessary habitat for native species in the face of changing environments and associated threats is fundamental to biodiversity conservation, and to justify their continued long-term designation and management. Our study indicates that hutias may require more intensive protection measures than solenodons, due to their apparently more localized distribution and restricted habitat associations. Management actions for both species should focus on preventing human settlement and encroachment within PAs and, in particular, improving protection of core areas of older high-quality forest, a move also likely to benefit many other native species on Hispaniola. With a considerable proportion of the Dominican Republic under strict protection, and escalating pressure on land outside PAs, these areas are likely to play an increasingly important role for securing the future of Hispaniola’s last remaining native land mammals.
